# mGem: Deciphering how polyomaviruses coexist with their hosts for a lifetime

**DOI:** 10.1128/mbio.02311-25

**Published:** 2026-01-05

**Authors:** James M. Pipas, Christopher S. Sullivan

**Affiliations:** 1 Department of Biological Sciences, University of Pittsburgh 171653, Pittsburgh, Pennsylvania, USA; 2 Department of Molecular Biosciences, LaMontagne Center for Infectious Disease, The University of Texas at Austin 196204, Austin, Texas, USA; Georgia Institute of Technology, Atlanta, Georgia, USA

**Keywords:** polyomavirus, latency, barcode, microRNA, cell cycle, small DNA virus

## Abstract

Small DNA tumor viruses such as polyomaviruses have evolved persistent, in some cases lifelong infections despite their compact genomes and host immune pressure. This review synthesizes historical and recent insights into the mechanisms underlying polyomavirus persistence and shedding, including dynamic host cell cycle regulation, viral non-coding control region modulation, and viral microRNA-mediated repression. We highlight modes of shedding consistent with concurrent latent/lytic and smoldering infections, discuss emerging evidence of reversible latency, and identify unresolved questions in viral-host interplay. Understanding these strategies is critical for managing viral reactivation and disease in immunocompromised patients and exemplifies the remarkable evolutionary success of polyomaviruses.

## PERSPECTIVE

It has been said that “Energy and persistence conquer all things” (Benjamin Franklin). Viral persistence refers to the ability of some viruses to establish long-term infections rather than being cleared by the immune response (1). While herpesviruses have a well-deserved reputation for lifelong latent and lytic infections, they are not unique in their ability to persist. Some small DNA viruses, such as polyomaviruses, with genomes 20–45 times smaller, also maintain chronic—and in some cases lifelong—infections, even in the face of an immune system dedicated to the eradication of invaders (2). How these enigmatic, small-genome viruses fly under the radar has only recently begun to be unraveled. Advances have highlighted an intricate interplay between viral transcriptional programs (3), microRNA (miRNA)-mediated regulation (3–5), host cell cycle cues (6–8), and a spectrum of persistent infection strategies, including smoldering (constant low-level replication and shedding) and episodic, high-output viral shedding (9). This review surveys historical milestones in polyomavirus persistence and presents emerging insights into the possibility of true latency and reactivation.

## THE POLYOMAVIRUSES: GENOME, HOST, AND DISEASE

Polyomaviruses were first identified through the ability of murine polyomavirus (muPyV) to induce tumors of diverse origin in laboratory mice, hence the name “poly-oma” (10). Of the approximately 14 known human polyomaviruses, at least four are associated with disease, predominantly in immunosuppressed individuals (11–13). The BK polyomavirus (BKV or BKPyV) is linked to nephropathy and kidney inflammation (14), and JC polyomavirus (JCV or JCPyV) causes progressive multifocal leukoencephalopathy (PML) (15). Merkel cell polyomavirus (MCPyV) is the only human PyV established to cause cancer (16). In immunocompetent hosts, most polyomaviruses, including BKV, JCV, and MCPyV, typically cause no pathology, establishing a long-term coexistence.

Modes of viral shedding often reflect tissue tropism: BKV and JCV dwell in and are shed from the kidney, while viruses such as MCPyV and Trichodysplasia spinulosa polyomavirus infect and shed from skin. Many studies of polyomavirus infection utilize infection of actively dividing transformed cells and in some cases utilize viruses such as simian virus 40 (SV40) strain 776 with heavily rearranged non-coding control regions (NCCRs) that have been adapted for robust laboratory lytic virus production (17). Thus, despite extensive cell culture and animal studies, the precise mechanisms by which these viruses target, persist, and disseminate in specific tissues remain elusive. The rise in transplantation and the aging population and associated immunosuppression underscore the urgency of understanding polyomavirus persistence and disease reactivation.

## LATENCY: FROM HERPESVIRUSES TO POLYOMAVIRUSES

**Fig 1:**
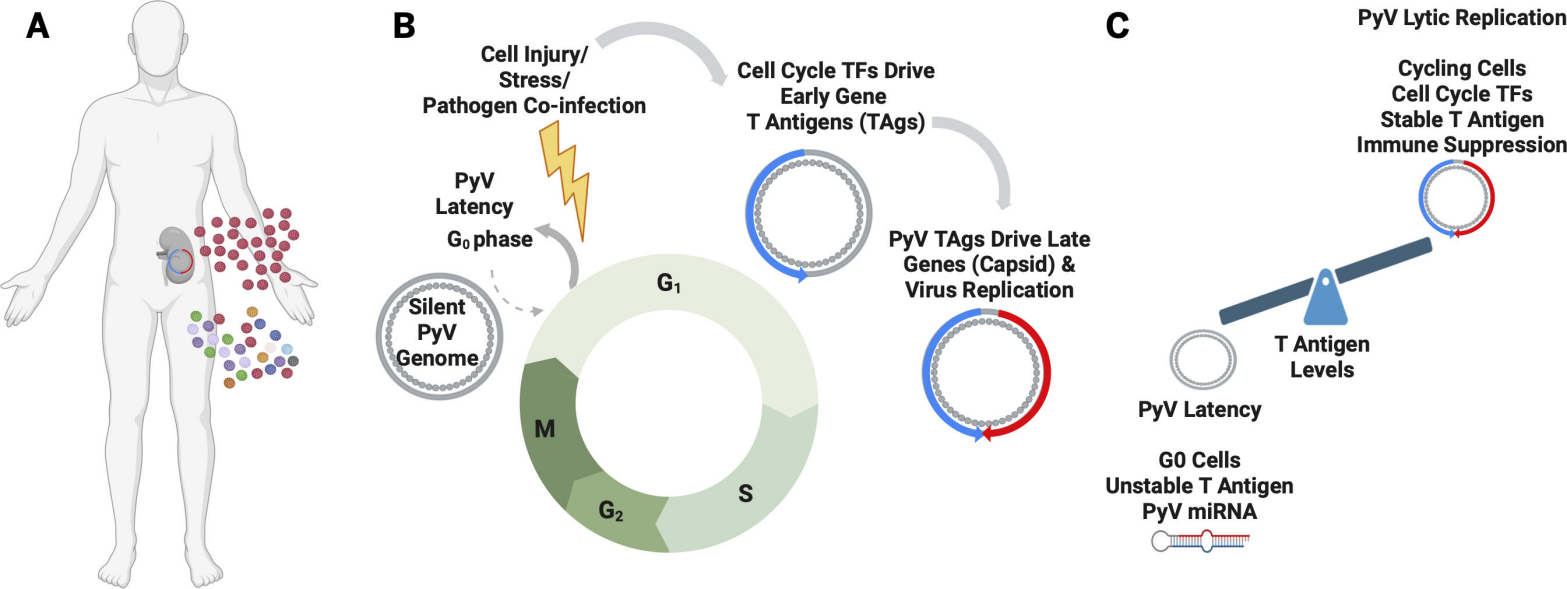
Hypothesis: polyomavirus (PyV) persistence is maintained by both smoldering and latent/lytic mechanisms, and the latent/lytic switch is controlled by differential large T-antigen levels driven by the transition from G0 to G1/S phases of the cell cycle. (**A**) Illustration of observed PyV shedding patterns: continuous low-level release suggests smoldering infection from multiple reservoirs, while intermittent high-level spikes indicate pronounced reactivation from one or a few latent reservoirs. (**B**) A model in which G0-phase cells serve as latent reservoirs. In this scenario, PyV genomes remain quiescent until host cells transition into the G1/S phase, triggered by events such as tissue damage. (**C**) T-antigen expression and stability. In G0 cells, low transcription, combined with unstable T antigen and viral miRNA, maintains viral silence despite occasional leaky transcriptional noise. In the G1/S phase, robust expression of stable T antigen occurs, supporting viral reactivation. Together, these data suggest the stage of cell cycle provides a toggle mechanism by which PyVs alternate between cryptic persistence and emergence from latency.

Latency, classically studied in herpesviruses, is one mechanism of viral persistence defined as an alternative, fully reversible genetic program resulting in non-productive infection that can transition to active, lytic replication (18). Proving true latency—where the same cell switches between silent and active genetic states—requires single-cell evidence that is currently lacking for small DNA viruses. Nonetheless, studies with BKV suggest that entry into and exit from persistence is tightly regulated by the host cell cycle (6, 7). For instance, renal proximal tubule epithelial cells infected with BKV that have low viral gene expression are more likely to be growth arrested (6), and BKV remains largely quiescent in G0-arrested cells but becomes transcriptionally active when these cells re-enter the cell cycle (7). The mechanism for this is unknown, but a plausible model is that cycling cells activate host transcription factors (19) that promote viral transcription. As just one example, Sp1 (20) is differentially activated by cell cycle progression and has known docking sites in NCCRs of diverse PyVs (21) (Fig. 1).

## POLYOMAVIRUS LATENCY AND PERSISTENCE *IN VIVO*

Longitudinal studies in humans reveal that most immunocompetent individuals episodically shed high levels of BKV or JCV in urine, followed by periods of cryptic (silent) infection (22–24). Some immunocompetent individuals, however, show continuous shedding, at least over the period of months (24). Large swaths of the adult human population (50%–90%) are seropositive for PyVs (including JCV, BKV, and MCPyV) (12) consistent with persistent infection and frequent reactivation of multiple PyVs. Immunosuppression, whether due to untreated HIV infection or immunosuppressive therapy, precipitates high levels of shedding and disease (2, 25, 26), confirming the critical role of the immune response in maintaining control of polyomavirus shedding.

Laboratory models, such as SV40, corroborate these patterns. SV40 can establish long-term kidney infections in its natural (rhesus macaque [27]) and experimental (hamster [28]) hosts, with viral replication escalating under experimental immunosuppression (29). Early mouse studies with murine polyomavirus by Dubensky and Villarreal—predating the polymerase chain reaction era—demonstrated widespread polyomavirus dissemination post-infection, with kidneys serving as long-term reservoirs consistent with urine as a key route for viral release (30). Villarreal and colleagues pioneered new technologies such as whole mouse *in situ* hybridization for viral nucleic acids and *in vivo* plasmid transfection (30–32). These studies swapped enhancer elements showing altered muPyV tissue tropisms, confirming the importance of the NCCR in the outcome of persistent infection. These authors were also among the first to propose different modes of persistent infection, including something akin to fulminant lytic, smoldering, and latent infections and that cellular division or kidney damage could promote PyV reactivation (31, 33). Overall, distinct infection phases have been observed for different PyVs, which may correspond to various modes of latency and reactivation.

Modern genetic barcoding of muPyV has revealed two main viral shedding patterns: a continuous persistent, “smoldering” background of multigenotype virus release and sporadic, high-output bursts of one or a few persistent reservoirs (9) evocative of reactivation from a latent state. Factors influencing these different patterns remain unknown, but likely candidates include differing cell-type-specific transcription factor repertoires (19) as well as systemic cues (e.g., pregnancy hormones [34, 35], type I interferons [36–38], and adaptive immune suppression [2, 4, 39]) and local stimuli (e.g., tissue damage/stress [40], inflammation [41], and pathogen coinfection [42, 43]) leading to cell cycle progression.

## THE ROLE OF THE NCCR IN PERSISTENCE

The polyomavirus genome, a simple circular double-stranded DNA of approximately 5 kb, consists of early genes (tumor antigens, or “T antigens”) and late genes (capsid proteins), both governed by an enhancer and opposing promoters within the NCCR (44). The NCCR integrates host transcription factor inputs and presumably orchestrates the switch between quiescent and active viral states. Rearrangements in the NCCR are particularly common in immunosuppressed patients and are linked with increased viral replication and disease, notably in BKV nephropathy and PML from JCV (45). However, “archetype” viruses—those without NCCR rearrangements—are most commonly transmitted, suggesting evolutionary selection for attenuated replication in immunocompetent hosts.

Historically, polyomavirus large T antigens are well characterized for their ability to inhibit pRB family members and free E2F driving cells into cycle (13). It is an interesting twist that it now appears that cell cycle transcription factor regulators such as Sp1 (upregulated in G1 phase) and others, via the NCCR, likely play pivotal roles in regulating expression of the viral early genes. In addition, large T-antigen stability has been implicated in the latency-productive infection switch (46–50). Combined evidence suggests a model where cellular stress or proliferation cues can upregulate NCCR activity and/or T-antigen stabilization, leading to increased T-antigen levels that promote a feed-forward loop of virus gene expression resulting in viral reactivation and renewed shedding (Fig. 1).

## POLYOMAVIRUS miRNA: BALANCING REPLICATION AND PERSISTENCE

Several well-studied polyomaviruses encode miRNAs that post-transcriptionally regulate viral gene expression, primarily by targeting early viral mRNAs (51–57). These miRNAs are thought to prevent excessive antigen production, minimize immune recognition, and stabilize latency (58). In this model, the viral miRNA plays a supporting role to the NCCR by limiting leaky T-antigen expression, thus curbing viral replication and helping the virus persist undetected by cytotoxic T-cell lymphocytes. Such a model is appealing in its simplicity as prevention of transcriptional noise is a known common function of host miRNAs (59). Mutant muPyV polyomavirus lacking the miRNA gene shows decreased replication in early times post-infection in mice. However, consistent with the above model, at later times when bulk virus shedding is dominated by high shedding events of one or a few genomes (9), the miRNA mutant displays significantly enhanced shedding (4), highlighting a fine-tuned balance between silent smoldering persistence and regulated reactivation.

## DO POLYOMAVIRUSES UNDERGO TRUE LATENCY?

Current evidence points to both persistent, low-level (smoldering) infections and punctuated, high-output viral shedding events (9), hallmarks of potential latency and reactivation. While human/mouse shedding patterns, cell culture findings, and virus barcode studies are consistent with the existence of a reversible latent/lytic switch, definitive demonstration of true latency (i.e., single-cell genetic program reversibility) awaits further research. Regardless, polyomaviruses are highly successful at establishing and maintaining persistent infections, a testament to their evolutionary adaptation despite their compact genomes.

## REMAINING QUESTIONS AND FUTURE DIRECTIONS

Key unresolved issues include the following.

- How do different polyomaviruses regulate the transition between silent persistence and robust lytic reactivation, particularly in response to cell cycle progression and cellular stress?
- What systemic and local cues—such as nephrotoxicity, coinfection, and pregnancy hormones—trigger reactivation?
- What is the precise contribution of the adaptive and innate immune responses in restraining reactivation and disease, especially in immunosuppressed settings, and how do these differ in smoldering versus latent/lytic modes?
- Do polyomaviruses have true latency, and if so, what are the alternative viral genetic programs and what enforces and reverses them?
- Do the same cell types serve as reservoirs to smoldering shedding and latent/lytic infection, and what fraction of infected cells are producing virus in either mode?
- Do differences in cell cycle stage directly contribute to latent/lytic switch, or is it simply correlative with another process?
- Can therapeutic interventions focused on preventing PyV reactivations be developed to prevent viral disease in transplant and other high-risk patients?

Emergent models, including barcode viral genetics and physiologically relevant cell culture systems mimicking G0-arrest and cell cycle entry, in tandem with mouse models, promise to clarify these mechanisms.

## CONCLUSIONS

Polyomaviruses, with only ~5 kb of DNA, achieve remarkable evolutionary success. From their origins deep in evolutionary time (60) to their ability to persist in humans across both lifespans and population migrations (61–63), polyomaviruses embody the concept of coexistence through life. Their ability to toggle between quiescent and active states relies on an elegant integration of host signals, viral regulatory regions, protein stability, and viral miRNAs. Dissecting the subtle choreography of polyomavirus persistence and reactivation is critical—not only for fundamental virology but also for managing opportunistic diseases in immunosuppressed individuals. As polyomaviral molecular tools and experimental models have advanced, we stand poised to unravel the silent yet influential journey of these elegant copassengers through life.
